# Sex-dependent differences in avian malaria prevalence and consequences of infections on nestling growth and adult condition in the Tawny pipit, *Anthus campestris*

**DOI:** 10.1186/s12936-016-1220-y

**Published:** 2016-03-22

**Authors:** María Calero-Riestra, Jesus T. García

**Affiliations:** Instituto de Investigación en Recursos Cinegéticos (IREC), CSIC-UCLM-JCCM, Ronda de Toledo s/n, 13071 Ciudad Real, Spain

**Keywords:** Avian malaria, *Haemoproteus*, *Plasmodium*, Tawny pipit, *Anthus campestris*, Infection, Host age, Host sex, Nestling growth, Nest predation

## Abstract

**Background:**

Parasites play pivotal roles in host population dynamics and can have strong ecological impacts on hosts. Knowledge of the effects of parasites on hosts is often limited by the general observation of a fraction of individuals (mostly adults) within a population. The aim of this study was to assess the prevalence of malaria parasites in adult (≥1 year old) and nestling (7–11 day old) Tawny pipits *Anthus campestris*, to evaluate the influence of the host sex on parasite prevalence in both groups of age, and explore the association between infections and body condition (adults) and growth (nestlings).

**Methods:**

Two hundred Tawny pipits (105 adults and 95 nestlings) from one Spanish population were screened for avian malaria parasites (*Haemoproteus* and *Plasmodium*) using the polymerase chain reaction (PCR)-based methods. Body condition (body mass against a linear measure of size) was measured in adults and growth rate (daily mass gain) was calculated for nestlings.

**Results:**

The overall prevalence of infection was 46 %. Sixteen different mitochondrial cytochrome *b* haplotypes of *Plasmodium* spp. and one *Haemoproteus* spp. haplotype were found. Malaria parasites were equally prevalent in nestlings and adults (45 and 46 %, respectively). Males were more likely to be infected by parasites than females, and this sex-bias parasitism was evident in both adults and nestlings. Furthermore, a lower daily mass gain during nestling growth in males than in females following infections were found, whereas the effect of infections on body condition of adults was detrimental for females but not for males.

**Conclusions:**

Age-specific differences in physiological trade-offs and ecological factors, such as nest predation would explain, at least in part, the observed host sex and age-related patterns in Tawny pipits.

## Background

Theoretical and empirical work on a variety of host-parasite systems has demonstrated the central role that parasites play in many important aspects of host biology [1]. Prevalence of arthropod-borne parasites in wild animal populations reflects a balance between the exposure of hosts to the infective stages of a parasite, and the success of the parasite in infecting the hosts [2]. This balance between host exposure and parasite success is partially influenced by the immunological capability of the host, which is known to be sensitive to traits such as sex, age, physiology, body condition and reproductive effort [3–7]. Avian malaria parasites of genera *Plasmodium* and *Haemoproteus* are blood parasites that occur regularly in wild populations and are one of the most popular model systems for studying host-parasite co-evolution [5, 8]. This type of parasites had attracted considerable attention as disease causes a variety of adverse effects on hosts [9–17], including the extinction of naive host populations [18]. Although the infections caused by these parasites can also persist in hosts for years causing no direct effects or only mild effects on fitness [9, 12, 19–21], recent studies have revealed that long-term chronic infections can produce important hidden costs for hosts [17].

The vast majority of studies exploring malaria impacts on wild avian hosts have been focused on adults, which inevitably restrict the ability to understand properly the consequences of infections for hosts. For example, during the acute phase of malaria infection many individuals were naturally removed from the population by dying [21], which makes really difficult to assess what type of parasites are involved and the real damage they caused at the population level. In this context, one interesting approach is to use nestlings as research focus. New-borns represent an attractive target for malaria parasites because of their low immunological and behavioural defences, which are relatively immature at hatching [22, 23]. Development of the immune system begins post-hatching and continues during the growth period, when birds are able to mount a significant immune response to blood parasites [24]. Therefore, age determines host cell maturation and the capacity to resist against parasites and support infections (reviewed in [25]). A naive immune system is known to increase susceptibility to parasitism and, therefore, those parasites that can take advantage of the immunologically naive hosts to increase its recruitment and survival rates should be favoured [25]. In birds, the immunological naive hosts (nestlings) are highly available to vectors only during early stages of development, whereas for much of the remaining year the host population consists of individuals with experience of prior infection. Moreover, infected nestlings may face at this stage a trade-off between allocating resources to growth or to keep them as immune enhancers for fighting parasites or diseases [26–29]. Thus, adults and nestlings should be considered altogether in order to better understand how parasites (in terms of species composition and prevalence) impact globally on a population. Despite its importance, surprisingly little is known about the avian malaria infections during the crucial phase of nestling growth as compared to adult phase.

Furthermore, parasite prevalence in vertebrates is often different between males and females [30, 31], which has been mainly attributed to sex-specific host characteristics, such as the endocrine-immune interactions. Mounting evidence indicates that sex hormones influence the immune system [32–34]. Androgens, primarily testosterone, can suppress cell-mediated and humoral immunity in males, whereas oestrogen can suppress cell-mediated immunity while boosting humoral immunity in females [32]. In birds, evidence suggests that an effective immune system is costly, and that there are trade-offs among investment in immune function and other physiological and ecological aspects during reproduction [35–37]. This may result in differences in parasite prevalence between the sexes [4, 38]. There is, therefore, a need to assess what role, if any, sex-related trade-offs between investment in immunity and other aspects of reproduction play in mediating acquisition of parasites, and if costs of parasite infections are different for each sex.

In this study, nestling and adults from a wild population of the open-ground nestling passerine Tawny pipit (*Anthus campestris*) were used as a novel host-parasite system to explore: (1) how malaria parasites are distributed among adults and nestlings, (2) the influence of the host sex on parasite prevalence in both groups of age, and (3) the association of parasite prevalence with adult body condition and nestling growth.

## Methods

### Ethical consideration

All research was carried out within Spanish standard requirements (project license reference JCCM/PAC 06-137) and the guidelines of the University of Castilla-La Mancha (CCM). All methods were approved by the University of Castilla-La Mancha ethical committee for Animal experimentation (CEEA) and permission to capture and manipulate birds was obtained from the Organismo Autonomo de Espacios Naturales de Castilla-La Mancha (permission numbers: OAEN/SVSIA/avp_10_153, and DGPF/08031701). Birds were caught under Spanish standard requirements (bird ringing license numbers: SEO/BirdLife 520038 and 530351).

### Host species, study areas and sampling method

The Tawny pipit is a small (~28 g) migratory passerine of the family *Motacillidae*. It is a widespread summer visitor of open habitats throughout Eurasia [39]. Tawny pipits are sexually monomorphic, with slightly larger males than females (mean ± SD; wing length 94.0 ± 3.42 and 88.0 ± 2.32 mm for males and females, respectively; [39]). Males and females have different roles during breeding; while nest attendance and care for nestlings are exclusive to females, males are responsible for territory defence [40]. The open-cup nests are constructed directly on the ground [41]. Clutch size varies between three and five eggs, and nestlings are altricial (range time in the nest between 8 and 11 days) [40].

The study was conducted during the reproductive seasons of 2008, 2009, and 2010 in a study area with approximately 2.5 km^2^ located in Valeria, central Spain (Cuenca province, 39º 48′ N, 2º 10′ W, 1090 m above sea level). The study site is located in a flat terrain, where the species’ density is about 15 breeding pairs/km^2^. Climate is Mediterranean (annual rainfall is 750 mm and mean annual temperature 16 °C) and the prevailing habitat consists on natural shrub steppe vegetation (*Rosmarinus officinalis*, *Thymus* spp. and pastures dominated by *Brachypodium phoenicoides*) with scattered cereal crops.

Nest visits were performed at intervals of 3–5 days throughout the breeding season (May–July). A total of 105 adult birds were captured in their territory around nests (males, *n* = 63) or at nest when feeding nestlings (females, *n* = 42). Adults were measured (wing length and mass), ringed with a metal ring to avoid resampling, and 5–10 μl of blood was collected from the jugular vein. Additionally, 95 nestlings (48 males and 47 females) from 28 nests were sampled twice at nest, when they were 1–3 day-old and when they were 7–11 day-old. During the first visit, all nestlings were marked with different waterproof colours on tarsus and feet to allow individual recognition. Wing length was measured with a ruler (accuracy 0.5 mm) and body mass with a digital balance (accuracy 0.01 g) in the two visits. During the last visit all nestlings were banded with a metal ring and 2–5 μl of blood was taken. Tawny pipits were classed in two age groups for analyses: adults (≥1 year old) and nestlings (7–11 day old). Blood samples were preserved in ethanol until DNA analyses. All birds sampled were captured and handled with the corresponding permissions of both regional and national Spanish authorities.

### Genetic characterization of parasites

DNA was extracted from blood samples using ammonium acetate/ethanol precipitation methods [7], and diluted to a working concentration of 25 ng/μl. All birds were molecularly sexed by amplifying introns of the CHD1Z/W gene [42]. For the detection of parasites we used the nested polymerase chain reactions (PCR) protocol used by Waldenström [43], which amplify a fragment of the mitochondrial cytochrome b gene of *Plasmodium* spp. and *Haemoproteus* spp. parasite genera. Each PCR included two positive samples of infected birds to verify the proper functioning of the reaction and a negative (ddH_2_O) to control for the presence of false positives. A volume of 2.5 µl of each final reaction was evaluated on 2 % agarose gels and stained with ethidium bromide. Pre- and post-PCR work was performed with different material and in different laboratory sections to avoid contamination. The protocol was repeated three times to confirm negative results. Samples with positive PCR reactions were cleaned up using Exonuclease I and Shrimp Alkaline Phospatase (Fermentas) and sequenced using the forward PCR primer and the Big Dye Terminator Kit (Applied Biosystems). DNA sequences were obtained using an ABI 3130XL Automated Sequencer (Applied Biosystems). Data were processed with the ABI PRISM1 Sequencing Analysis Software v3.7 (Applied Biosystems). New parasite lineages were sequenced twice to ensure the accuracy of the sequences.

### Phylogenetic analysis

Sequences were edited manually using Bioedit 7.0.5.3 [44]. All new haplotypes were deposited in GenBank (accession numbers: JF279937–JF279958 and KF747759–KF747764). The taxonomic identity of each haplotype was inferred by assessing the phylogenetic affinities with published sequences from GenBank that were reliably identified as morphological species of *Plasmodium* and *Haemoproteus*, as compiled in the MalAvi database [45]. Multiple infections (more than one parasite haplotype in the same individual) were identified by the presence of double peaks on the electropherograms [46]. Phylogenetic relationships among parasite haplotypes were estimated by Bayesian inference using MrBayes 3.1.2 [47]. The GTR+I+G model of molecular evolution was identified by jModelTest [48] as the most appropriate for the dataset. Two simultaneous runs of four Monte-Carlo Markov Chains (MCMC) were conducted over 5 million generations, sampled every 100 generations. The posterior probability distribution of the 50 % majority rule consensus tree was calculated after discarding the first 25 % generations as the burn-in period. The phylogenies were visualized using FigTree v1.3.1 [49].

The genetic divergence between different parasite haplotypes was calculated using the Tajima-Nei distance model computed in the software MEGA4 [50].

### Statistical analysis

The effects of host sex and age on infection probability were analysed by means of generalized linear/nonlinear mixed models (GLMMs) with a binomial distribution for our dependent variable (infection status: infected or uninfected), and a logit link function. The model included year as random factor and age, sex, and their interaction as fixed effects. Date of sampling was incorporated as a continuous predictor to account for its possible effect on the infection probability. Date was calculated as the number of days from 1st April within each study year until the day of sampling. Individuals carrying multiple infections were treated as ‘infected’ in this analysis. Difference in the proportion of sexes and age cohorts infected by each parasite clade was tested using Fisher’s exact test (two-tailed).

The effect of infection status on body condition was estimated for adults by means of linear mixed models (LMMs). The model included body mass as dependent variable and wing length as continuous predictor (to account for differences in size between individuals). Infection status (infected or uninfected), sex and their interaction were included as fixed factors. The model included also the year of study as a random factor and date of sampling as continuous predictor.

Additionally, we performed LMMs to investigate whether infection influence nestling growth (growth increments for mass and wing length) using the two measurements taken on each nestling during its nest stage. The rate of daily mass gain during growth was expressed as [(mass at second visit—mass at first visit)/number of days between first and second visit], and the rate of daily wing length increment was expressed as [(wing length at second visit—wing length at first visit)/number of days between first and second visit]. Two independent models were built, with mass gain or wing length increment as dependent variables, infection status (infected or uninfected), sex and their interaction as fixed factors, age of nestling and sampling date as continuous predictors, and year and nest identity as random factors.

All analyses were performed using R 2.14 [51] (R Core Team 2013). Specifically, package *lme4* [52] was used to fit LMMs and GLMMs, and package *phia* [53] to perform post hoc analyses. Means and parameter estimates are reported together with their standard errors.

## Results

In total, 92 out of the 200 birds screened were infected with malaria parasites, which represents an overall prevalence of 46 % (Table 1). Multiple infections (3.3 %) and unresolved (poor-quality) sequences (2.2 %) were excluded from further analyses. Sequences from 86 *Plasmodium* (99 %) and one *Haemoproteus* (1 %) species were recorded, yielding 16 unique *Plasmodium* and one *Haemoproteus* cytochrome *b* haplotypes. The most common parasite haplotype in Tawny pipits was BIC33 (31 %), followed by BIC39 (16.1 %), BIC8 (13.8 %), BIC29 (12.6 %) and BIC32 (11.5 %). Other haplotypes were detected at low rate (less than 3.0 % of all infections). A Blast search (GenBank’s basic alignment search tool) was performed for our cytochrome b haplotype dataset, identifying six *Plasmodium* haplotypes that had been previously recorded in other avian hosts: BIC29 (KF747759), BIC32 (KF747760), BIC33 (KF747761), BIC36 (KF747762), BIC37 (KF747763) and BIC39 (KF747764). The remaining 11 parasite haplotypes had not been previously described, and showed between 0.23 and 1.87 % of genetic divergence with respect to any other *Plasmodium* or *Haemoproteus* lineage recorded, to date, in avian hosts.

**Table 1:** Prevalence (in brackets) of malaria parasites in adult and nestling, male and female Tawny pipits from 2008 to 2010 in Valeria, Spain

Years	Adults				Nestlings			
	Male		Female		Male		Female	
	Uninfected	Infected	Uninfected	Infected	Uninfected	Infected	Uninfected	Infected
2008	24	8 (0.25)	16	2 (0.11)	15	5 (0.25)	14	2 (0.12)
2009	7	12 (0.63)	8	7 (0.46)	5	8 (0.61)	12	7 (0.37)
2010	0	12 (1.0)	2	7 (0.77)	1	14 (0.93)	4	8 (0.66)
Total	31	32 (0.51)	26	16 (0.38)	21	27 (0.56)	30	17 (0.36)

### Clustering of parasite lineages

**Fig. 1:**
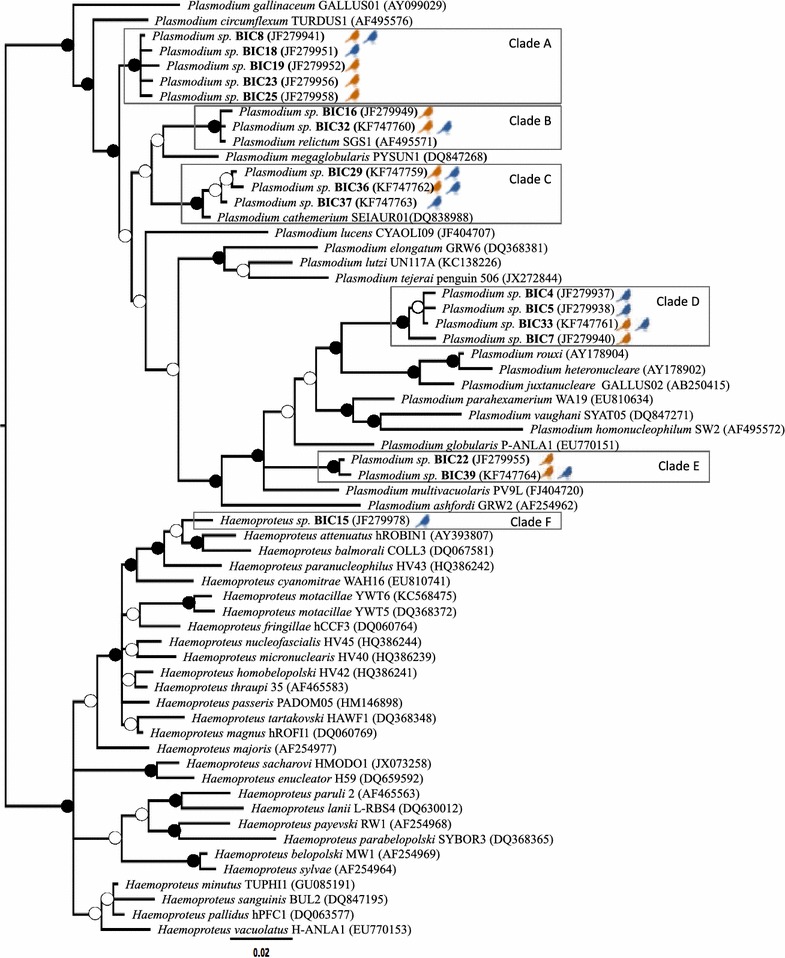
Phylogenetic relationships between the Cytochrome b sequences obtained in the study and those of known Plasmodium and Haemoproteus species (represented by their accession number). The tree was built on cytochrome *b* parasite haplotypes found in this study (*bold*), with respect to lineages previously found in other avian hosts. *Black* and *white circles* indicate, respectively, nodes with posterior probability >90 %, and between 50 and 90 %. Tawny pipit haplotypes are grouped in clades that should correspond to different parasite species, and their presence in adults (*blue bird icon*) and nestlings (*orange bird icon*) is also shown

The phylogenetic tree that resulted from Bayesian analyses is shown in Fig. 1. Morphospecies nomenclature was tentatively assigned to smaller cladistic groupings within the *Plasmodium* and *Haemoproteus* genera based on the affiliation and bootstrap support of branches with lineages published previously.

Five distinct *Plasmodium* clades (clades A–E; Fig. 1) were found, with genetic distances between haplotypes within clades ranging from 0.2 to 0.7 % (mean = 0.44 %, S.D. = 0.2 %), whereas genetic divergence between these clades varies between 3.4 and 8.5 % (mean difference = 6.4 %, S.D. = 2.1 %). Of the five clades, only two can be associated to a known parasite morphospecies (clade C: *Plasmodium cathemerium*, and clade B: *Plasmodium relictum*). One single *Haemoproteus* clade (clade F) was detected, which included only one haplotype isolated from Tawny Pipits (BIC15) that did not contain sequences of any of the *Haemoproteus* morphospecies previously described.

### Variation in parasite prevalence

The prevalence of malaria infections in the Tawny pipit population studied did not show significant variation with date of sampling (Table 2). Overall, there were no significant differences between the infection status of the two age groups, being the proportion of infected adults and nestlings 0.45 and 0.46, respectively (see also Table 1). However, Tawny pipit females have a lower prevalence of malaria than do males in the 3 years of study (Tables 1, 2). The proportion of infected females and males was 0.37 and 0.53, respectively, and this pattern is consistent across ages, as supported by the non-significant interaction sex × age (Table 2).

**Table 2:** Generalized linear mixed model estimates

Response variable	Estimate	Statistic value (Z)	*P* value
Sampling date	0.02326	1.380	0.1665
Age	−0.40358	−0.778	0.4350
Sex	2.03322	2.130	*0.0332*
Sex × age	−0.85431	−0.793	0.4270

Infected adult (age) and female (sex) served as a reference groups. Statistically significant results are shown in italics

At parasite-clade level, avian malaria clades A–E (see Fig. 1) found in our study area were all present in both adults and nestling, whereas clade F (*Haemoproteus*) was only retrieved from adults. The prevalence of clade A in Tawny pipits was 8.0 % (n = 200), with no significant differences between age classes (adults: 4.76 %, nestlings: 11.58 %; Fisher exact test, two tailed, P = 0.11). The prevalence of clade B was 5.5 % (adults: 7.62 %, nestlings: 3.16 %, P = 0.22). The prevalence of clade C was 7.0 % (adults: 10.48 %, nestlings: 3.16 %, P = 0.053) whereas the prevalence of clade D was 15 % and similar between the two age classes (16.19 % in adults and 13.68 % in nestlings, P = 0.69). Finally, the prevalence of Clade E was 7.5 % (adults: 4.76 %, nestlings: 10.53 %, P = 0.18).

There were significant differences between the prevalence of clades B and C according to sex, being both clades more frequent in males than in females (clade B: males 12.0 %, females 1.12 %, Fisher exact test, two tailed, P = 0.02; clade C: males 9.0 %, females 1.12 %, P = 0.02). There were no statistical differences between males and females for clades A, D and E (P > 0.05).

### Infection status and body condition in adults

**Fig. 2:**
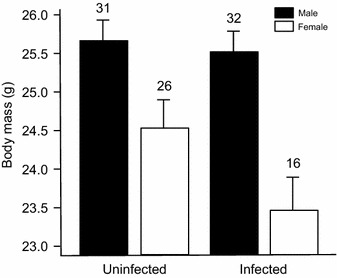
Effects of avian malaria infections on adult body condition. Host adult body mass after controlling by wing length (fitted means + SE from the model, see “Methods” section). The *figure* shows the interaction between infection status and sex in the linear mixed model (see “Methods” section). Sample size indicated above *error bars*. Other variables in the model are described in “Results” section

Overall, body mass was positively related with wing length (*t* = 2.70, *P* = 0.008), and was larger in males than in females (*t* = 1.94, *P* = 0.055; Fig. 2). Infected individuals were in poorer condition than uninfected ones (*t* = −2.91, *P* = 0.004), and the significant interaction (infection × sex: *t* = 2.00, *P* = 0.048) indicates that the effect of infection status on body mass was significant for females (post hoc: $\chi_{1}^{2}$ = 8.49, *P* = 0.007) but not for males ($\chi_{1}^{2}$ = 0.23, *P* = 0.63) (Fig. 2). There was no support for a significant effect of date of sampling on body mass (*t* = −0.78, *P* = 0.44).

### Infection status and growth in nestlings

**Fig. 3:**
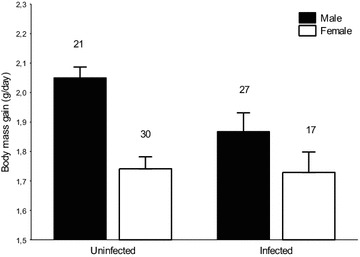
Effects of avian malaria infections on nestling growth. Variation in body mass gain (grams per day) in relation to infection status and sex of nestlings. Data are expressed as fitted mean + SE from the model (see “Methods” section). Sample size indicated above *error bars*. Other variables in the model are described in “Results” section

Sex of nestlings accounted for most of the variation in daily mass gain, and this variation was statistically significant (daily body mass gain = 1.90 ± 0.05 g. for males and 1.72 ± 0.05 for females, *t* = 5.48, *P* < 0.0001). Overall, infected nestlings gain weight at the same rate as uninfected ones (*t* = −0.035, *P* = 0.97). Interestingly, the interaction between sex and infection status is statistically significant (*t* = −2.29, *P* = 0.025) because infected males had a significant lower mass gain than uninfected males (post hoc: $\chi_{1}^{2}$ = 7.23, *P* = 0.014) whereas females did not ($\chi_{1}^{2}$ = 0.00, *P* = 0.97, Fig. 3). Neither age of nestling (*t* = −1.38, df = 51.58, *P* = 0.17) nor date of sampling (*t* = −0.11, df = 21.34, *P* = 0.91) was significantly related to daily mass gain.

Wing length increment was higher for males than for females (males: 5.18 ± 0.08 mm, females: 4.87 ± 0.09 for females, *t* = 3.52, *P* = 0.0008). Differences in wing length increment among infected and uninfected individuals were not significant (*t* = 1.03, *P* = 0.31), and the interaction between sex and infection status was also not significant (*t* = −0.51, *P* = 0.61). Date of sampling was not significantly related to wing length increment (*t* = 0.07, *P* = 0.97), whereas age of nestling is marginally significant in the model (*t* = −1.98, *P* = 0.053).

## Discussion

The present study adds data on host-parasite relationships for a so far non-investigated passerine species, the Tawny pipit. Host sex-related differences affecting prevalence of avian malaria, but no evidence of age-related differences, were reported here. Tawny pipit males were more likely to be infected by malaria parasites compared to females, and this sex-bias parasitism was evident in both adults and nestlings. Avian malaria parasites were also prevalent in 7–11 day-old nestlings, consistent with other reports [54]. Most importantly, infected males showed lower daily mass gain during nestling growth than infected females, whereas infections at adulthood were associated with lower condition in females than in males.

The effect of host age on parasite prevalence is a controversial topic in avian ecology. On one hand, the number of host-parasite encounters and the accumulation of infections over time means that we can expect a pattern of higher parasite prevalence in adults than at early stages of life [55, 56]. On the other hand, the low immunological and behavioural defences against parasites of nestlings compared to adults suggest the opposite pattern, with higher expected prevalence in nestlings than in adults [57, 58]. Few studies have explicitly compared avian malaria infections between nestlings and adults in the wild, since the prepatent period (when the parasite is developing in the host tissues and absent from blood) is often longer than the nestling period of most bird species (see [59]). Results from Tawny pipit supported the hypothesis that the naïve immune system makes nestlings highly susceptible to developing infections. The age-pattern of malaria infection found in this study indicated a rapid accumulation of parasites at the age at which an individual is first susceptible to infection. Considering the age of nestlings studied (7–11 days) and that prepatent periods for avian species of *Plasmodium* and *Haemoproteus* range from 2 days to several months [60], it is likely that some of the nestlings considered as uninfected in this study might be truly infected, but the disease has not yet reached the blood (prepatent stage) at the time of sampling. If so, the real parasite prevalence in nestlings can be underestimated, and the age-prevalence curve would reflect a decreasing incidence of malaria with age [58] typical of a parasite-mediated viability selection pattern [61, 62]. A reduction of prevalence with age should merely indicate the loss (death) of infected individuals from the population [58, 61], or that parasites were cleared from hosts or entered a latent stage (remaining usually in host tissues) later in life [63, 64]. Unfortunately discrimination among these possibilities is not possible in the present study, but the topic merits further research as they can reveal patterns that may allow quantification of rates of parasite transmission, parasite-induced host mortality, and development of host resistance.

Regarding the processes whereby infections occur, age-specific differences in physiological trade-offs would explain, at least in part, the observed pattern [58]. Nestlings hatch when parasite prevalence peaks in vector populations [65, 66], so they are expected to rapidly acquire numerous parasite lineages present in the breeding area. Maturation of the immune system in birds may take several weeks after hatching and, therefore, investment in immune defence may come at the expense of investment in somatic growth (review in [67]), which may be especially patent for species with determinate growth (i.e. growth ceases before chicks leave the nest) like passerines. Moreover, in species or populations at higher risk of nest predation the trade-off between investment in immune defence and investment in growth may be particularly important, as predation is one ecological factor that exerts strong selection on growth rates in passerines [68, 69]. Ground-nesting species living in open environments in the Mediterranean region are under strong predation rates [40, 70], and previous work conducted on the same Tawny pipit population has shown that nest predation is a key factor driving the species’ reproduction [40]. As a consequence, nestlings of altricial or semi-altricial species, such as Tawny pipits, should reach adult size faster than precocial species [71], indicating that growth is compressed into a shorter period fuelled by a strong evolutionary pressure to leave the nest as soon as possible in order to avoid or minimize predation [72]. Therefore, nestlings may prioritize growth over development of immune function, causing high disease incidence in this age group despite the short time of exposure to parasites. The high prevalence found in nestlings, together with the fact that most parasite haplotypes were found infecting both nestlings and adults points towards transmission of these parasites in the European breeding grounds of Tawny pipits. The only exception was the *Haemoproteus* haplotype, which was detected only in adults. This suggest that parasites of this clade could have been transmitted in winter quarters (i.e. Africa), as proposed in the case of many other *Haemoproteus* and *Plasmodium* haplotypes for which the occurrence of suitable vectors in Europe could be currently absent [73]. Alternatively, perhaps, the prepatent period for *Haemoproteus* spp. described in the literature (between 11 days and 3 weeks) diminishes the probability of detection in the nestling age cohort.

Differences between males and females in prevalence and/or intensity of infection, in particular male-biased parasitic infections, are often observed in nature [6, 31, 35, 74–76]. However, sex-bias in parasitism is not universal and consistent, and often varies between and within host-parasite systems [3, 58, 77]. In Tawny pipits, males had higher parasite prevalence than females in both age cohorts, a pattern compatible with gender differences in susceptibility to parasites according to biological differences between host sexes [78]. In the avian malaria system, host sex is considered as a potentially important source of variation in both prevalence and cost of parasites for hosts [78]. The proximate explanations for sex-bias in parasitism may be caused by many different factors that revolve around two hypotheses, which are not mutually exclusive: 1) certain hormones have an immunosuppressive effect upon the host defence against pathogens, and 2) because of morphological and/or behavioural differences, one sex has a greater likelihood of being parasitized by differential exposure to vectors [35, 79]. In Tawny pipits, males and females do not differ substantially in mass or colour [80] and their home ranges overlap within territories, which were probably not large enough (3.5–12.1 ha; [81]) to explain differences in vector exposure between males and females. Although behavioural differences cannot be excluded as a potential explanation, there is overwhelming evidence that sex-associated hormones can directly influence the susceptibility of each sex to infections [35]. For example, testosterone has immunosuppressive effects in some species, leading to increased susceptibility of males to parasite infections [82]. However, while the immunosuppressive effect of sex-linked hormones is a well-recognized phenomenon in adulthood [83, 84], that could explain the results found in adults, its rationale in the case of nestlings is not clear, as differences in susceptibility to parasite infection might not be attributable directly to sexual differences in circulating testosterone levels [85]. Firm conclusions cannot be drawn about why males were more infected than female nestlings, but the results found on differential growth between sexes (discussed below) should shed some light on this issue.

Male and female nestlings leave the nest at the same time; therefore, for a species where males are slighter larger in wing size than females [80], males may need to grow faster than their smaller sisters, as supported by the higher wing growth rate of male nestling compared to females found in this study. The growth strategy of small breeding-ground passerines under strong nest-predation rates may be due to the need to leave the nest as soon as possible, which depends on their ability to fly and, thus, the development of the wings. If male nestlings need to allocate more resources to growth and less to prevent infections than females this could result in increased parasite prevalence in males as compared to females. In a comparative study with amphibians, Johnson et al. [86] showed that amphibian species with rapid growth were more susceptible to infections and pathology than species with slow growth. Within birds, selection for growth compromises the immune function in lines of commercial poultry (meta-analysed by van der Most et al. [87]). Although the increment in wing length was higher for male than for female nestlings, wing-growth strategies in both sexes seem to be unaffected by parasite infections. As a faster development of wings can facilitate both escape from predators and survival outside the nest, growth of body components that reduces the risk of predation may be relatively prioritized over mass in species at higher risk of nestling predation [71]. In contrast, there is a clear sex-biased growth strategy in relation to the rate at which individuals gain body mass under malaria infestation. The advantage of ‘being a male’—attaining higher weight and larger biometric size than sisters—, and the competitive strength related to this [88] becomes a handicap under parasite infestation, when the selective pressure for a faster growth is strong. In our study, the impact of parasites in term of mass gain is particularly important in the fastest growing sex [89], which support this idea. Unfortunately, the potential incidence of a reduced condition during growth on posterior mortality rates cannot be evaluated here, but several authors have demonstrated this kind of relationships [90, 91]. Therefore, in species/populations that evolved under strong (nest) predation pressures, such as Tawny pipits [40], the impact of malaria parasites might ultimately influence offspring mortality rates.

Once adulthood is reached, selection pressures changed, and the trade-offs between immune system and investment in reproduction makes females more susceptible to the effect of malaria parasites than males. In our species model, females make greater investment than males in terms of time and energy during breeding, since females are solely responsible for incubation and chick rearing, which included the search for food and feeding chicks [40]. As a result, Tawny pipit females show a continuous decline in body condition during the breeding season of around 27 % of initial mass [92]. Here, infection was associated to a reduced host condition for females but not for males, supporting the view that infections by malaria parasites cause sex-related differences in host body condition, presumably as a result of increased investment in reproduction by females compared to males. However, it is also possible that host with reduced body mass may be less likely to fight off parasites or their vectors. Further work is clearly needed to conclusively evaluate the link between reproductive effort in both sexes and the effect of parasites acquired by Tawny pipits.

## Conclusions

Most studies only consider adults when investigating avian malaria parasites and as a result infection in nestlings is under-represented in the literature. This study has focussed not only in adults (in terms of parasite prevalence and association with body condition), but also considered infections of these parasites in nestlings (prevalence and effects on nestling growth). This study has demonstrated high prevalence of avian malaria parasites in nestlings (7–11 day-old), comparable to adult prevalence, and sex-related differences in both age groups. Both the acquisition of these parasites as well as the consequences of parasites for individuals depends on host sex and age. Trade-offs between investment in immune system and investment in other tasks (reproduction in adults and growth in nestlings) may explain the results found, and some ecological factors (nest predation) could exacerbate the effects of malaria parasites for avian hosts at the population level.
